# Electrospun Nanofiber-Based Viroblock/ZnO/PAN Hybrid Antiviral Nanocomposite for Personal Protective Applications

**DOI:** 10.3390/nano11092208

**Published:** 2021-08-27

**Authors:** Abdul Salam, Tufail Hassan, Tooba Jabri, Shagufta Riaz, Amina Khan, Kanwal Muhammad Iqbal, Saif ullah Khan, Muhammad Wasim, Muhammad Raza Shah, Muhammad Qamar Khan, Ick-Soo Kim

**Affiliations:** 1 Nanotechnology Research Group, Department of Textile and Clothing, Faculty of Engineering and Technology, National Textile University Karachi Campus, Industrial Area Korangi, Karachi 74900, Pakistan; ab.salam@ntu.edu.pk (A.S.); hassan.tufail@ntu.edu.pk (T.H.); 2 International Center for Chemical and Biological Sciences, H.E.J. Research Institute of Chemistry, University of Karachi, Karachi 75270, Pakistan; toobaasif137@gmail.com (T.J.); kanwalmuhammadiqbal43@gmail.com (K.M.I.); raza.shah@iccs.edu (M.R.S.); 3 Functional Textile Research Group, Department of Textile Engineering, National Textile University, Faisalabad 37610, Pakistan; Shaguftariaz84@gmail.com; 4 Department of Chemistry, National Textile University, Faisalabad 37610, Pakistan; aminakhan1649@gmail.com; 5 Department of Textile Engineering, Balochistan University of Information Technology Engineering and Management Sciences, Quetta 87100, Pakistan; saifk1543@gmail.com; 6 Key Laboratory of New Materials and Modification of Liaoning Province, School of Textile and Materials Engineering, Dalian Polytechnic University, Dalian 116034, China; Muhammad_wasim786@yahoo.com; 7 Division of Frontier Fiber, Institute of Fiber Engineering, Interdisciplinary Cluster for Cutting Edge Research (ICCER), Faculty of Textile Sciences, Shinshu University, Tokida 3151, Ueda, Nagano 386 8567, Japan

**Keywords:** hybrid composites, polymer fibers, face masks, polymers, nanocomposites, Viroblock, zinc oxide

## Abstract

Designing novel antiviral personal protective equipment (PPE) is crucial for preventing viral infections such as COVID-19 in humans. Here, we fabricate an electrospun nanofiber-based Viroblock (VB)-loaded polyacrylonitrile (PAN)/zinc oxide (ZnO) hybrid nanocomposite for PPE applications. Five different concentrations of Viroblock (0.5%, 1.5%, 2.5%, 3.5%, and 5%) were added to PAN/ZnO solution and loaded for electrospinning. The developed samples reflected antibacterial activity of 92.59% and 88.64% against *Staphylococcus aureus* and *Pseudomonas aeruginosa* bacteria, respectively, with 5% VB loading. Moreover, a significant reduction in virus titer (37%) was observed with the 5% VB/PAN/ZnO nanofiber sheet. Hence, VB-loaded PAN/ZnO nanofibers have great potential to kill enveloped viruses such as influenzas and coronaviruses and could be the ideal candidate for the development of nanofiber-based PPE, such as facemasks and surgical gowns, which can play a key role in the protection of frontline health workers and the general public in the COVID-19 pandemic.

## 1. Introduction

In recent times, the world has progressed in various domains, but viral infections persist to grow day by day, which not only affects human lives but also the global economy [1]. Many viral infection outbreaks have been caused by the Nipah virus [2], coronavirus [3], dengue virus [4], and influenza virus [5]. Recently a novel coronavirus (COVID-19) has caused an acute pandemic, claiming the lives of millions of people throughout the world [6]. Viruses are small infectious substances that can reproduce by attacking the host cell. Enveloped viruses (such as influenza and coronavirus), in which the capsid is enveloped by a lipid bilayer membrane, are considered to be a more dangerous form of viruses. In contrast to non-enveloped viruses, enveloped viruses can survive on surfaces for several days and remain infectious [7], which causes serious viral infections. There are six different stages of the pathogenesis of viral infections: (i) point of entry for virus attachment, (ii) diffusion into a host cell, (iii) virus uncoating, (iv) replication and protein synthesis, (v) assembly of the naked capsid, and (vi) release of the virion [8]. Aerosol droplets are the main source of transfer of infectious disease, and in such circumstances, personal protective equipment (PPE) such as protective gowns, face masks, and face shields can play a pivotal role in reducing the spread of viral disease [9,10,11]. Though most personal protective equipment can electrostatically repel or physically block the viruses and bacteria, any resident bacteria or virus that can survive on the surface of the PPE could cause contamination during disposal or reuse. Considering the current COVID-19 pandemic, due to the shortage of PPE supplies, reuse and sterilization of PPE are still challenging [12]. Fabric-based face masks are considered to be an effective shielding material against aerosol particles [13]; however, the contamination of the fabric surface can be still a big issue. To address this issue, researchers are working on surface functionalization of PPE by using different antimicrobial and antiviral agents, such as ZnO, TiO_2_, WO_3_, CuO, and Ag nanoparticles, to disinfect and deactivate the residing pathogens present on the surface of PPE. However, the limited affinity of the aforementioned functional nanomaterials towards textile-based PPE is a great challenge. Researchers are overcoming this challenge by using different binders or in situ growth of nanomaterials on the surface of the fabric. Seino et al. developed antiviral textile through the immobilization of silver nanoparticles on the surface of cotton fabric by using a radiochemical process [14]. Norazi et al. functionalized polyester fabric by in situ growth of SiO_2_ nanoparticles for antibacterial and antifungal protective clothing [15]. Shaheen et al. developed in situ zinc oxide nanoparticles on the surface of cotton fabric to enhance durability [16]. Several researchers used a binder to increase the durability of particles attached to personal protective equipment. Naka et al. reported that metal ions such as Mg, Ag, Cu, Zn, Al, and Ca impart the antiviral effect to textile material [17]. Imai et al. reported that Cu–zeolite-coated textile showed excellent antiviral activity against avian influenza virus H5 due to the presence of Cu^+2^ ions as compared to zeolite-coated textile [18]. Sumit et al. used a shellac and copper nanoparticle-based spray-assisted nanocoating on nonwoven polypropylene fabric to fabricate photoactive face masks possessing a hydrophobic surface with self-cleaning properties. Under solar light irradiation, more than 70% free radicals have been reported, which leads to the disruption of the membranes of nanosized virus-like particles [19]. Nevertheless, in the case of in situ growth of nanomaterial on PPE surfaces, particles can easily peel off during washing [20], which may cause serious health issues for the end-user, while binder functionalization diminishes comfort and sensorial properties of the personal protective clothing [21]. To fix these problems caused due to in situ growth and the use of binders, electrospinning technology is considered to be one of the best candidates for the production of durable PPE (face masks, surgical gowns, etc.), because in this case, antibacterial and antiviral materials are directly doped in the spinning solution and become part of the final produced electrospun nanofiber sheet. Moreover, the exceptional surface functionality of nanofibers corresponds with high surface area [22,23]. Cui et al. embedded CuO nanoparticles in hydrophobic polyvinylpyrrolidone (PVP) polymer by the electrospinning method. Oxygen plasma was used to etch the CuO nanoparticles in the PVP surface. Antiviral activity of the CuO embedded nanofibers was tested against the H1N1 virus, and results revealed that 70% of viruses were inactivated after 4 h of contact [24]. Rashid et al. reported that the addition of silver nanoparticles to polyaniline polymer resulted in the enhancement of both antiviral and antibacterial activity [25]. Ji et al. conducted their study on the development of side-by-side nanofibers for biomedical applications by using the electrospinning technique. One side of the prepared nanofibers was composed of ZnO nanoparticles, while the other side was composed of silver nanoparticles. They found that the resultant nanofibers showed excellent antibacterial activity against Gram-positive and Gram-negative bacteria [26]. In another study, Kim et al. developed an electrospun nanofiber-based membrane for wound dressing applications. That particular membrane was modified by using silver nanoparticles assisted by polydopamine (PDA). The resultant silver nanoparticle functionalized membranes were biocompatible and had antibacterial activity against Gram-positive and Gram-negative bacteria [27]. Chowdhury et al. developed a bio-based antiviral mask by using the electrospinning technique and claimed that licorice root extract loaded into Polyvinyl alcohol (PVA) electrospun nanofibers had the potential of killing the bacteria and viruses due to the presence of glycyrrhetinic acid and glycyrrhizin [28]. Multifunctional poly(methyl methacrylate) (PMMA) nanofibers decorated with ZnO nanorods and silver nanoparticles provide significant protection against Gram-positive and Gram-negative bacteria as well as protect against viruses like coronavirus and influenza viruses [29].

Herein, considering the current COVID-19 pandemic situation and excessive demand for protective clothing, we are the first to present a robust methodology to develop efficient antiviral and antibacterial electrospun nanofibrous membranes by incorporating HeiQ Viroblock (VB) and ZnO nanoparticles in a polyacrylonitrile (PAN) electrospun solution. Zinc oxide is a multifunctional material with exceptional properties (photocatalysis, antibacterial properties, ultraviolet (UV) protector) [30,31], but in this specific study, it acted as an antibacterial agent. HeiQ Viroblock is a white viscous liquid that encompasses two main components, namely a vesicle component and a silver component [32]. The vesicle component contains cosmetic grade liposome, which is responsible for the direct depletion of the envelope of the viruses, while the silver component contains nanoscale silver particles that have the potential to destroy the bacteria and viruses [33,34,35]. The developed electrospun nanofibers have the potential to be used for protective clothing (surgical gowns, face masks) against enveloped viruses (influenza, coronaviruses) and bacteria (Gram-negative and Gram-positive).

## 2. Materials and Methods

### 2.1. Materials

All the chemicals were purchased from different sources and used in this study without further additional purification. Polyacrylonitrile (PAN, MW = 150,000 g/mol) was used as the main precursor material for the fabrication of nanofibers due to its better fouling properties, high chemical stability, and high mechanical strength [36]. Polyacrylonitrile was purchased from Sigma Aldrich, Seoul, Korea. Zinc oxide nanoparticles (ZnO NPs, 50–60 nm), acting as an antibacterial and UV protective agent, were procured from Sigma Aldrich, Seoul, Korea. Viroblock (combination of silver and lipid vesicle), acting as an antiviral as well as an antibacterial agent, was purchased from HEIQ Concord, NC, USA. N,N-Dimethylformamide (DMF, MW = 73.1 g/mol, 99.5%, Reagent Grade), used as a solvent, was purchased from DaeJung, Seoul, Korea.

### 2.2. Preparation of Pristine PAN, PAN/ZnO, and VB-Loaded PAN/ZnO Electrospinning Solutions

In this study, a total of seven different solutions were prepared. Pure PAN solution (10 wt%) was prepared by dissolving 1 g of PAN powder in 9 g of N,N-dimethylformamide (DMF) solvent. The solution was allowed to stir magnetically on the magnetic stirrer for about 10 h at a stirring speed of 500 rpm to form a homogeneous solution. For the PAN/ZnO composite solution, 5 wt% ZnO nanoparticles were added and allowed to stir magnetically at 500 rpm for approximately 15 h. Moreover, different solutions of PAN/ZnO loaded with VB (different concentrations, namely 0.5 wt%,1.5 wt%, 2.5 wt%, 3.5 wt%, and 5 wt%) were prepared by allowing the composite solution to stir magnetically at 500 rpm for approximately 23 h. In all the prepared VB-loaded ZnO/PAN composite solutions, the concentration of ZnO nanoparticles remained constant.

### 2.3. Fabrication of Electrospun Nanofibers

**Figure 1:**
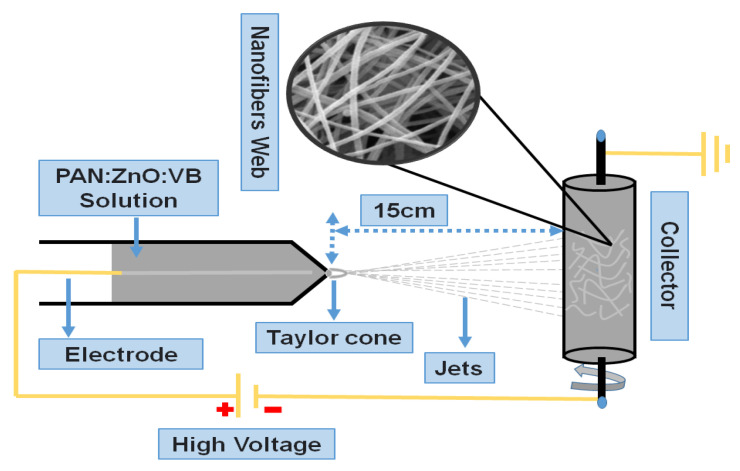
Illustration scheme of fabrication of antiviral nanofibers.

**Figure 2:**
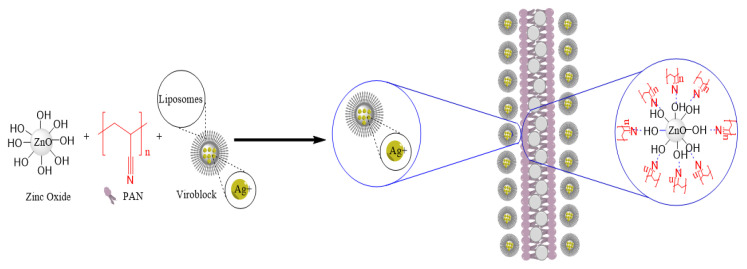
Chemical reaction for the fabrication of antiviral nanofibers.

For the fabrication of pure PAN electrospun nanofibers, PAN solution was poured in 10 mL of a disposable syringe, which was connected to the pipette tip. A copper wire acting as a positive electrode was fitted inside the syringe. The solution in the form of nanofibers was drawn through the tip of the syringe under the applied voltage of 17 kV. These extruded fibers were collected on the aluminum foil wrapped on the negative charged rotating cylinder (collector). The distance between the collector and tip of the syringe was kept at 15 cm while maintaining the flow rate of 1 mL/h. Similarly, this process was repeated for the fabrication of PAN/ZnO and VB-loaded PAN/ZnO electrospun nanofibers. The illustration scheme and possible reaction of fabrication of electrospun nanofibers are shown in Figure 1 and Figure 2, respectively.

## 3. Characterization

### 3.1. Surface Morphology

The surface morphology of the prepared electrospun nanofibers was analyzed by using a scanning electron microscope (SEM) (Model: JSM-5300, Tokyo, Japan), by applying 12 kV voltage. ImageJ software (version 1.49) was used to calculate the average diameter of electrospun nanofibers by taking 200 random measurements from each prepared sample [22].

### 3.2. Transmission Electron Microscope (TEM) Analysis

To check the uniform distribution of nanoparticles and VB in electrospun nanofibers, TEM analysis was used. High-resolution TEM (JEOL ARM 200F, Tokyo, Japan) was used to capture the TEM images at a working voltage of 200 kV [37,38].

### 3.3. Chemical Interaction (FTIR)

The chemical interaction between PAN, ZnO NPs, and Viroblock (VB) was analyzed by Fourier Transform Infrared (FTIR) (PerkinElmer, Billerica, MA, USA). Spectra of all the prepared samples were recorded from 400 cm*^−^*^1^ to 4000 cm*^−^*^1^. The spectra were recorded with 128 scans with a resolution of 4 cm*^−^*^1^.

### 3.4. Antibacterial Activity Protocol

Quantitative antibacterial analysis of prepared electrospun nanofibers was conducted by following the AATCC-100 standard [39]. According to this standard, the samples were shaken in the bacterial suspension of known concentration, and a reduction in the bacterial activity was noted in standard time. Antibacterial activity was characterized by measuring the viable microbial cells present on the nanofibers. To determine the change of *Pseudomonas aeruginosa* and *Staphylococcus aureus* contact with nanofibers, the bacterial plate method was applied for various time points.

The bacterial culture broth was incubated overnight. The triplicate method [40] was used to analyze the antibacterial activity of the electrospun nanofibers, while the average value was reported. Samples of nanofibers were cut into small circular pieces and submerged in the ethanol (75%) and dried before inoculation. Bacterial culture was diluted with cold 0.9% (*v*/*v*) silane solution (2 × 10^5^ CFU/mL). Moreover, the bacterial inoculum was cooled down to slow down the bacterial growth. The nanofibers were inoculated with 200 μL of cold and diluted bacteria and afterward placed in 20 mL of cold silane solution and shaken for a minute to detach the bacteria. The treated mixture was serially diluted in a 1:10 ratio and spread on a media agar plate for incubation at 37 °C for 24 h. The rate of inhibition or antibacterial efficiency was calculated by using the following formula:

(1) $$R=\frac{A-B}{A}\times 100$$

here, 

R = antibacterial efficiency (reduction percentage);

*A* = no. of bacteria in the broth detached from the membrane after inoculation at 0 h;

*B* = no. of bacteria recovered from the broth after 24 h of contact with a treated membrane.

### 3.5. Assessment of Antiviral Activity

The antiviral activity was evaluated by the following procedure. Firstly, a sample suspension of disinfectant was mixed with an infectious dose (ELD50) of avian influenza (AI) virus with an equal volume 1:1 (1 mL disinfectant + 1 mL virus infectious dose) and vortexed for 5 s followed by incubation for 2 h at 2–8 °C in a shaking incubator. After that, 100 µL of each mixture was inoculated via the chorioallantoic sac route with 8 embryonated eggs of each group. The hole-in-eggs were then sealed with molten wax and incubated for 48–72 h and were daily monitored to examine the viability of embryos. After inoculation, eggs were chilled to 24 h to settle down the Red Blood Cells (RBCs), eggshells were removed from the top of the eggs, and 10 mL allantoic fluid (AF) was harvested and centrifuged at 5000 rpm for 5 min for the removal of debris from AF. Supernatant was collected to check the virus growth. To check the virus titers, 50 µL of saline in a U-shaped bottom 96-well microtiter plate was used; 50 µL of tested AF was added in the first column of the 96-well plate, and subsequent wells were added typically with two folds up to the eleventh column. The final well served as a negative control with no virus. After serial dilution, 0.7–1% of chick RBCs were added to each well and mixed gently. The plates were incubated for 30 min at room temperature. Following the incubation period, the assay was analyzed by agglutination reaction and button formation [41].

## 4. Results and Discussion

### 4.1. Surface Morphology of Electrospun Nanofibers

**Figure 3:**
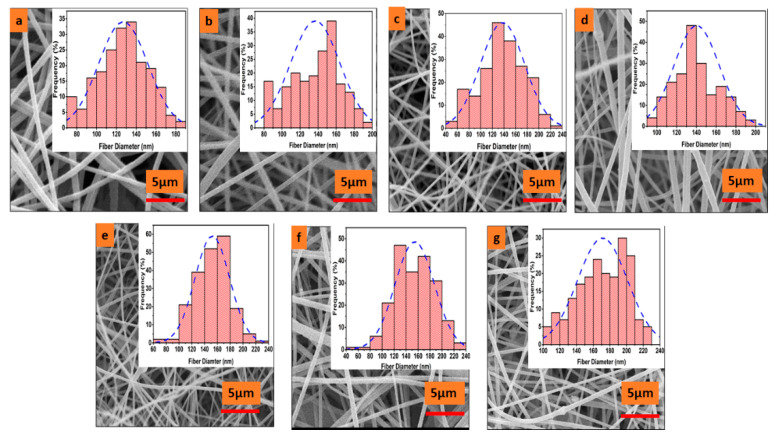
Surface morphology and average diameter of prepared VB-loaded ZnO/PAN electrospun nanofibers, (**a**) pristine PAN electrospun nanofibers, (**b**) ZnO/PAN electrospun nanofibers, (**c**) 0.5% VB-loaded ZnO/PAN electrospun nanofibers, (**d**) 1.5% VB-loaded ZnO/PAN electrospun nanofibers, (**e**) 2.5% VB-loaded ZnO/PAN electrospun nanofibers, (**f**) 3.5% VB-loaded ZnO/PAN electrospun nanofibers, (**g**) 5% VB-loaded ZnO/PAN electrospun nanofibers.

Figure 3 shows the surface morphology and average diameter of prepared electrospun nanofibers. Figure 3a represents the pristine PAN electrospun nanofibers having a length that ranges from hundreds of micrometers to millimeters. It can be observed that the surface of pristine PAN nanofiber is smoother and well-oriented compared to the other samples. By the incorporation of ZnO nanoparticles, the surface of nanofibers became courser and rougher. This roughness may be due to the agglomeration properties of ZnO nanoparticles on the surface of PAN electrospun nanofibers, as shown in Figure 3b–g. The diameter of prepared electrospun nanofibers was calculated by using Image J software. Two hundred measurements for each prepared sample were taken from different areas, and the average diameter was calculated by plotting histograms. As seen in Figure 3a, a minimum average diameter of 127 ± 24.8 nm was observed in the case of pristine PAN electrospun nanofibers. The maximum average diameter of 171 ± 29.88 nm was observed in the case of 5% loaded ZnO/PAN electrospun nanofibers, as shown in Figure 3g. In the case of Figure 3b–g, the observed average diameters were 136, 138, 140, 152, and 154 nm, respectively. It could be observed that as the concentration of VB increased, the average diameter of the electrospun nanofibers continued to increase. This could be explained by the fact that by incorporation of ZnO nanoparticles and VB in the polymer solution, the viscosity of the solution continued to increase, which may have resulted in a steadier and courser diameter of electrospun nanofibers [42,43].

### 4.2. TEM Analysis of Electrospun Nanofibers

**Figure 4:**
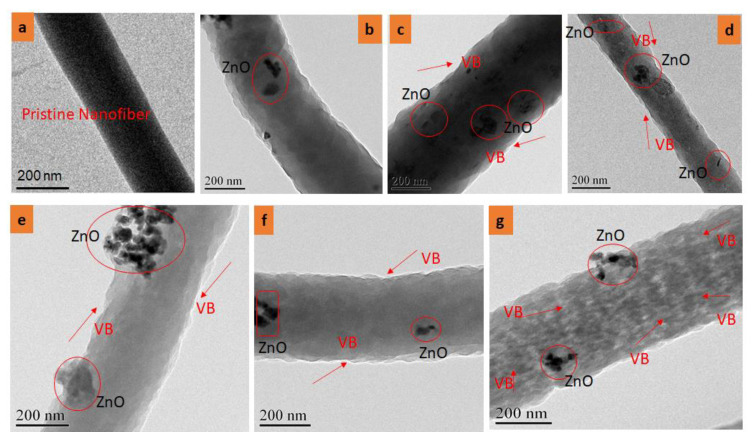
TEM analysis of Prepared VB-loaded ZnO/PAN electrospun nanofibers: (**a**) pristine PAN electrospun nanofibers; (**b**) ZnO/PAN electrospun nanofibers; (**c**) 0.5% VB-loaded ZnO/PAN electrospun nanofibers; (**d**) 1.5% VB-loaded ZnO/PAN electrospun nanofibers; (**e**) 2.5% VB-loaded ZnO/PAN electrospun nanofibers; (**f**) 3.5% VB-loaded ZnO/PAN electrospun nanofibers; (**g**) 5% VB-loaded ZnO/PAN electrospun nanofibers.

To investigate the effect of ZnO NPs and VB on the morphology of pristine PAN, PAN/ZnO, and PAN/ZnO/VB nanofibers, TEM images were studied, as shown in Figure 4. It was confirmed that neat PAN nanofibers had a smooth surface, but the surface morphology of PAN/VB/ZnO nanofibers became rough and blackish spots appeared on the surface by the doping of VB in the spinning solution. It was also confirmed that there were NPs of ZnO at the surface of the PAN/ZnO/VB nanofibers. Figure 4g reveals that due to the maximum concentration (5%) of VB in the PAN/ZnO/VB nanofibers, the blackish shade was increased as compared to the low concentrations.

### 4.3. FTIR Analysis of Electrospun Nanofibers

**Figure 5:**
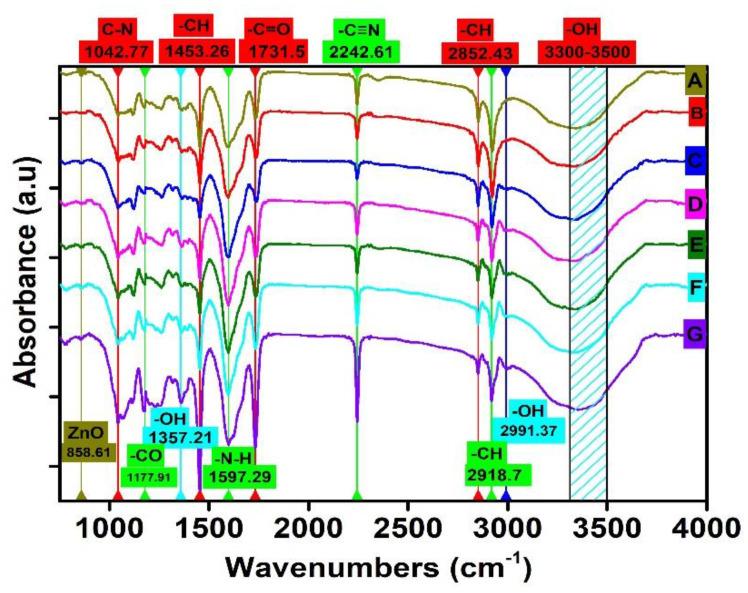
FTIR Analysis of VB-loaded ZnO/PAN electrospun nanofibers: (**A**) pristine PAN electrospun nanofibers; (**B**) ZnO/PAN electrospun nanofibers; (**C**) 0.5% VB-loaded ZnO/PAN electrospun nanofibers; (**D**) 1.5% VB-loaded ZnO/PAN electrospun nanofibers; (**E**) 2.5% VB-loaded ZnO/PAN electrospun nanofibers; (**F**) 3.5% VB-loaded ZnO/PAN electrospun nanofibers; (**G**) 5% VB-loaded ZnO/PAN electrospun nanofibers.

To understand the structure of prepared electrospun nanofibers, FTIR analysis was performed, as shown in Figure 5. In all spectra (a–g) there was broadband that appeared at wavenumbers of 3400 cm*^−^*^1^ to 3500 cm*^−^*^1^ corresponding to the –OH functional group. This band increased largely as ZnO and VB were added to the electrospun nanofibers. The –OH band had more depth in the case of spectrum B than spectrum A, which might be due to the hydrophilic nature of ZnO, which allowed more –OH radical to be absorbed onto electrospun nanofibers. However, by increasing the concentration of Viroblock, this band further increased, and the broadness of the band decreased, which might have been due to the presence of highly reactive silver ions (Ag^+^) in the VB that could attract more hydroxyl radicals from the atmosphere, as shown in (b–g). In all spectra, the characteristic peaks appeared at 2918.7, 2852.43, and 1453.26 cm*^−^*^1^, which corresponded to –CH group vibration and stretching due to alkene. The sharp peaks appeared at 1731.5 and 2242.61 cm*^−^*^1^ indicating C=O stretching and –C≡N stretching due to acrylamide [44]. The characteristics peaks in all spectra appearing at 1042.77, 1177.91, 1357.21, and 1597.29 cm*^−^*^1^ were related to C–H stretching, C=O stretching, –OH bending, and N–H bending, respectively. In all spectra (except a and b), a minute peak appeared at 2991.37 cm*^−^*^1^ corresponding to the –OH group, which might be due to the presence of silver ions (Ag^+^), as well as polar liposomes present in the Viroblock. These silver ions and liposomes tend to attract moisture from the atmosphere. In Figure 5B–G, the characteristic peak at 858.61 cm*^−^*^1^ was due to the vibrational bond of ZnO; however, this peak did not appear in the case of pristine PAN electrospun nanofibers [45]. We are confident that all the attractions between the PAN, Viroblock, and ZnO were physical, not chemical [46].

### 4.4. Antibacterial Activity of PAN/ZnO, PAN/ZnO-Loaded Viroblock Electrospun Nanofibers

**Figure 6:**
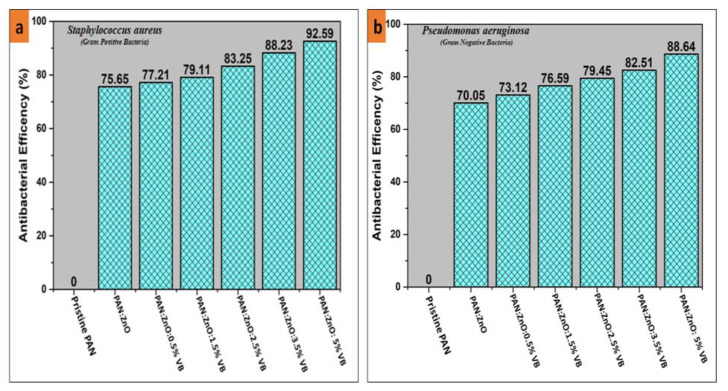
Antibacterial efficiency of pristine PAN electrospun nanofibers, ZnO/PAN electrospun nanofibers, 0.5% VB-loaded ZnO/PAN electrospun nanofibers, 1.5% VB-loaded ZnO/PAN electrospun nanofibers, 2.5% VB-loaded ZnO/PAN electrospun nanofibers, 3.5% VB-loaded ZnO/PAN electrospun nanofibers, and 5% VB-loaded ZnO/PAN electrospun nanofibers against (**a**)*Staphylococcus aureus* and (**b**) *Pseudomonas aeruginosa*.

**Figure 7:**
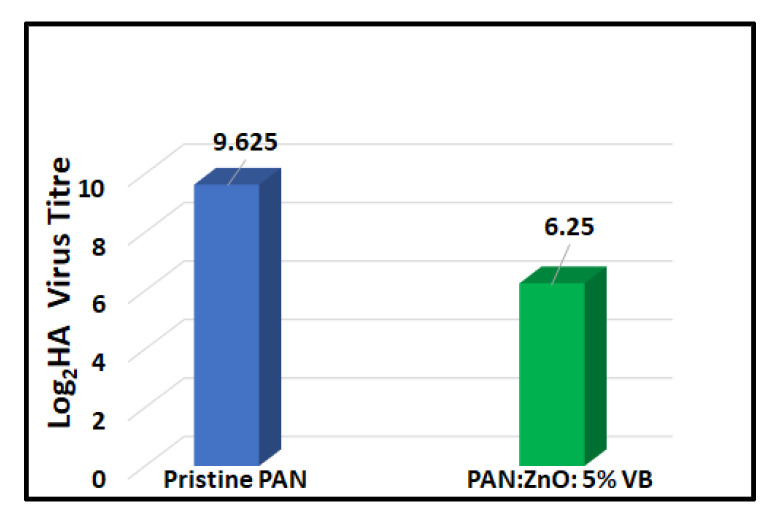
Antiviral activity of pristine PAN and 5% VB-loaded PAN/ZnO electrospun nanofibers.

Antibacterial activity of pristine PAN nanofibers, PAN/ZnO nanofibers, and PAN/ZnO nanofibers loaded with various concentrations of VB was observed by qualitative analysis followed by the AATCC-100 standard method. The antibacterial activity of all prepared electrospun nanofibers was tested against Gram-positive bacteria (*Staphylococcus aureus*) and Gram-negative bacteria (*Pseudomonas aeruginosa*). Figure 6a,b represents the antibacterial efficiency against Gram-positive bacteria and Gram-negative bacteria respectively. Pristine PAN electrospun nanofibers did not show any antibacterial activity. However, by the addition of the ZnO nanoparticles in the spinning solution, the PAN/ZnO electrospun nanofibers showed significant antibacterial efficiency against Gram-positive and Gram-negative bacteria. Figure 6 shows that PAN/ZnO electrospun nanofibers showed an antibacterial efficiency of 75.65% and 70.05% against Gram-positive *Staphylococcus aureus* and Gram-negative *Pseudomonas aeruginosa*, respectively, as metal oxide nanoparticles exhibited superior antibacterial properties, which suppressed the growth of both bacteria compared to the pure electrospun PAN nanofibers. Moreover, metal oxide nanoparticles provide reactive sites for the interaction of bacteria and nanofibers [47,48]. Additionally, metal oxides (ZnO) act as bacteriostatic agents [49] with positive charges that attract the negatively charged bacteria with a strong electrostatic force of attraction. As a result of this electrostatic interaction, the molecular structure of the phospholipid is broken, and as a result the cell membrane is also damaged, causing the death of bacteria [50,51]. With the addition of VB, the antibacterial efficiency of the electrospun nanofibers enhanced significantly against both Gram-positive bacteria as well as Gram-negative bacteria. VB contains silver particles that are potent antimicrobial as well as antiviral agents [34]. The addition of the VB in the spinning solution of the PAN/ZnO mixture resulted in the formation of ZnO–Ag heterostructure nanoparticles on the surface of PAN electrospun nanofibers. These heterostructure ZnO–Ag nanoparticles, when coming in contact with the bacteria, resulted in the disruption of the cell wall and cell membrane through a synergistic effect [52]. In addition, ZnO–Ag heterostructures inhibit the replication of DNA in the microbial cell by releasing Zn^+2^ and Ag^+^ ions [53]. Figure 7 shows that as the percentage of the VB increased, the antimicrobial efficiency further increased against both Gram-positive as well as Gram-negative bacteria, because by increasing the concentration of VB, more and more ZnO–Ag heterostructure came in contact with the bacterial cells, which led to significant enhancement of antibacterial efficiency. The maximum antibacterial efficiency shown by PAN/ZnO nanofibers loaded with 5% VB was 92.59% and 88.64% in the case of *Staphylococcus aureus* and *Pseudomonas aeruginosa*, respectively. Furthermore, Figure 6 shows that PAN/ZnO/VB hybrid composite electrospun nanofibers showed higher antimicrobial efficiency against Gram-positive than Gram-negative bacteria. This could be explained by one of possible three reasons. Firstly, the cell wall of Gram-negative bacteria is thicker than the cell wall of Gram-positive, and therefore Zn^+2^ or Ag^+^ ions can easily penetrate the cell wall of Gram-positive bacteria rather than Gram-negative-bacteria [54,55]. Secondly, in comparison to Gram-positive bacteria, Gram-negative bacteria are more resistant to reactive oxygen species (ROS) due to the complex permeable barrier of the cell wall [56]. Thirdly, the cell membrane of Gram-positive bacteria has a less negative surface charge; hence, negative reactive oxygen species (ROS) can easily pass through the cell membrane [57].

### 4.5. Antiviral Activity of Pristine PAN Nanofibers and 5% VB-Loaded PAN/ZnO Electrospun Nanofibers

**Figure 8:**
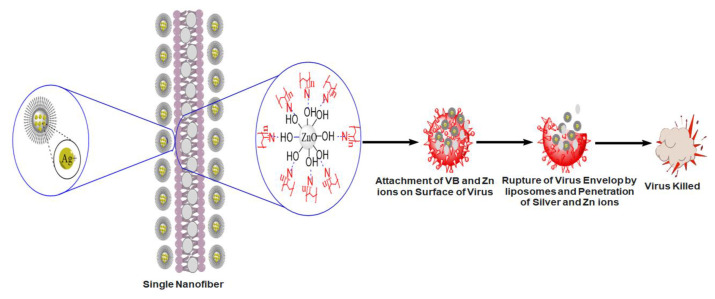
Schematic representation of antiviral activity of VB-loaded PAN/ZnO electrospun nanofibers.

Prepared electrospun nanofibers were tested for the antiviral activity against the avian influenza virus. PAN/ZnO nanofibers loaded with the maximum concentration (5%) of Viroblock were chosen for the antiviral activity, while pristine PAN nanofibers were used as a control sample. The plaque assay method was used to check the antiviral activity of prepared electrospun nanofibers. After three hours of contact with the virus, a significant decrease in the virus titers was observed, as shown in Figure 7, while in the case of pristine PAN nanofibers, there was negligible change in the virus titer. The average value of virus titer for the pristine electrospun nanofiber was 9.625, while in case of 5%VB-loaded PAN/ZnO electrospun nanofiber it was 6.25. From these results we can conclude that after three hours 5%VB-loaded PAN/ZnO nanofibers showed significant (37.5%) reduction as compared to the control sample, which showed a negligible reduction in the virus titer. The possible mechanism behind the high antiviral activity of VB-loaded nanofibers could be that VB contains silver ions and cosmetic-grade liposomes. Silver ions can kill viruses and bacteria due to their high surface energy and high surface area [34,35]. However, in the case of the influenza virus, the envelope of the lipid bilayer is present around the virus, which resists the penetration of the silver ions into the virus and thus protects the viral nucleocapsid from environmental stresses [58]. Due to this envelope, the efficiency of simple silver ions is not significant. To break the envelope of the virus, vesicle technology is used that contains cosmetic grade liposomes, which deplete the envelope membrane quickly so that the silver ions can attack the core of the virus and destroy the virus quickly. The possible mechanism of killing bacteria is shown in Figure 8.

## 5. Conclusions

This study is focused on the fabrication of antiviral nanofiber sheets by using the electrospinning technique. PAN/ZnO nanofibers were loaded with five different concentrations of Viroblock (acting as an antiviral agent). From the SEM analysis, it was confirmed that all the prepared electrospun nanofibers had smooth and bead-free surface morphologies. Uniform distribution of the ZnO and Viroblock in nanofibers was justified by using TEM analysis. FTIR analysis confirmed the physical interaction between PAN, ZnO, and Viroblock. Qualitative antibacterial analysis proved the strong antibacterial activity against both Gram-positive bacteria (*Staphylococcus aureus*) and Gram-negative bacteria (*Pseudomonas aeruginosa*). Further, higher antibacterial activity was recorded for Gram-positive bacteria (*Staphylococcus aureus*) than Gram-negative bacteria (*Pseudomonas aeruginosa*), corresponding to the thicker and more complex permeability of the barrier cell wall of the latter. It was also found that as the concentration of the VB increased, the antibacterial activity also increased significantly. The 5%VB-loaded PAN/ZnO electrospun nanofibers were used to check the antiviral activity against enveloped influenza virus. After three hours, a 37.5% reduction in virus titer was found in the case of 5%VB-loaded nanofibers as compared to pristine PAN nanofibers, which reveal the excellent antiviral activity of 5% VB-loaded nanofibers as compare to the pristine PAN nanofibers. From these results, it can be concluded that these VB-loaded PAN/ZnO electrospun nanofibers have great potential for the development of personal protective equipment such as facemasks and surgical gowns, which can play a key role in the protection of frontline health workers and the general public in the COVID-19 pandemic and onward.

## Data Availability

The data of this study are available from the corresponding author (M.Q.K.) upon reasonable request.
